# American Medical Association

**Published:** 1858-06

**Authors:** 


### AMERICAN MEDICAL SOCIETY.

### ELEVENTH ANNUAL MEETING.

*First Day.*—The Association met in the lecture-room of the Smithsonian Institution, and was called to order at a quarter past eleven o'clock, A. M., by Dr. Condie, of Philadelphia, when the chair was taken by the President, Dr. Paul F. Eve, of Nashville, Tennessee. Vice Presidents Brickinridge of Kentucky, Reese of New York, and Campbell of Georgia, were also on the platform; and at their tables were the efficient Secretaries, Drs. Foster, of Tennessee, and Semmes, of Washington.

Rev. Byron Sunderland, D. D., at the invitation of the President, offered an eloquent and appropriate prayer, invoking the blessing of Almignty God upon the Convention.

Dr. Harvey Lindsley, chairman of the committee of arrangements, then delivered an address of welcome.

The Secretary then called the roll by States, as it had been made out up to the commencement of the meeting, and the following number of delegates responded.

Maine 2, New Hampshire 8, Connecticut 18, Vermont 1, Massachusetts 40, Rhode Island 5, New York 73, New Jersey 25, Delaware 4, Maryland 24, District of Columbia 25, Virginia 8, North Carolina 8, South Carolina 10, Georgia 12, Alabama 1, Kentucky 9, Tennessee 7, Indiana 6, Illinois 12, Michigan 3, Iowa 3, Missouri 4, Ohio 14, California 1, American Medical Society of Paris 1, U. S. Navy 2.

Dr. Lindsley, chairman of the committee of arrangements, reported that it had been decided to hold but one business session each day, from 9 A. M., until 3 P. M. He also announced that the President of the United States would be happy to receive, with those members of the association who might call at the Executive Mansion at 8 o'clock in the evening, such ladies as may accompany them.

On motion, the association confirmed the appointment of Dr. J. M. Snyder to fill a vacancy in the committee of arrangements.

On motion, it was decided that a nominating committee of one from each State represented should be raised, the delegation of each State selecting its representative therein. A brief discussion upon the propriety of permitting the army and navy delegations to appoint separate members of the committee was decided by the President in favor of their having the privilege, and the decision was sustained by the association.

There was then a recess of fifteen minutes, during which the different delegations assembled in various parts of the lecture-room to choose their representatives in the committee. After the meeting was again called to order, the Secretary read the list as follows:

*Committee of Nomination.*—Job Holmes, Maine; Geo. H. Hubbard, New Hampshire; P. Pineo, Vermont; Ebenezer Alden, Massachusetts; Ashbel Woodward, Connecticut; J. Mauran, Rhode Island; H. D. Bulkley, New York; J. P. Colman, New Jersey; Isaac Hays, Pennsylvania; H. F. Askew, Delaware; Noble Young, District of Columbia; A. S. Payne, Virginia; W. H. McKee, North Carolina; William T. Wragg, South Carolina; Joseph P. Logan, Georgia; J. T. Hargraves, Alabama; R. J. Breckinridge, Kentucky; J. Berrian Lindsley, Tennessee; Wm. M. McPheeters, Missouri; George Mendenhall, Ohio; Calvin West, Indiana; A. H. Luce, Illinois; Zina Pitcher, Michigan; Thomas O. Edwards, Iowa; P. Smith, Maryland; O. Harvey, California, and Geo. Clymer, United States Navy.

On motion, Drs. Bohrer, of D. C., Flint, of New York, and Hargraves, of Alabama, were appointed by the President a committee on special essays.

Dr. David M. Reese, of New York, presented and read a written apology for having recommended for a position in Blockley Hospital, Philadelphia, Dr. McClintock, who had been expelled from the association for a violation of the ethics and the etiquette of the profession, by publishing a work on "domestic medicine," &c.

On motion of Dr. Condie, of Philadelphia, the apology was accepted, and ordered to be entered upon the minutes.

Dr. Bryan, of Philadelphia, who had also recommended Dr. McClintock, made a verbal adoption of Dr. Reese's apology, the reception of which was warmly debated. Dr. C. C. Cox, of Maryland, opposed, and Dr. Condie advocated the reception. Dr. A. B. Palmer, of Michigan, moved the previous question on a motion to refer the subject to a committee, which was lost. The apology of Dr. Bryan was then accepted.

The President then delivered, in a clear voice and with a pleasing oratorical effect, his Annual Address.

On motion, the thanks of the association were voted to the President for his able and instructive address, a copy of which was solicited for publication.

Dr. Grafton Tyler, of Georgetown, D. C., chairman of the committee on prize essays, reported that the essays received were three in number, each of which had been examined with great care; considering, first, the intrinsic merits of each essay, and their merits in relation to each other. The first prize was awarded to "an essay on the clinical study of the heart sounds, in health and disease," bearing the motto: "*Clinica clinice demonstrandum.*" The second prize was awarded to "an essay on vision and some of the anomalies as rendered by the opthalmiscope," bearing the motto: "*Dux hominum medicus est.*"

Dr. Tyler then proceeded to open the sealed envelopes bearing the above named mottoes, and containing the names of the writers of the essays. The first was written by Dr. Austin Flint of Buffalo, New York; and the second by Dr. Montrose A. Pallen, of St. Louis, Missouri. This is the second time Dr. Flint has won the distinguished honor, and the third time that it has been awarded to Buffalo since the association was organized, eleven years ago.

On motion the report of the committee was accepted and adopted. Drs. Flint and Pallen were then invited to give *resumes* of their essays, which they did.

Dr. Lindsley, from the committee of arrangements, then presented on invitation from Dr. Nichols to visit the Insane Asylum, and another from Rev. Mr. McGuire to visit Georgetown College.

On motion of Dr. Hamilton, of New York, these invitations were accepted, and the thanks of the association were returned therefor.

On motion of Dr. Lindsley, the Hon. Doctors Fitch, of Indiana, Chaffee, of Massachusetts, Clawson and Robbins of New Jersey, and Shaw, of North Carolina, members of Congress, and Dr. Peter Parker, ex-commissioner to China, were elected "members by invitation," and requested to participate in the proceedings of the association.

On motion, Assistant Surgeon Frederick A. Rose, of the British Navy, who so nobly volunteered his services on board the United States ship Susquehanna at Port Royal, and who came in her to New York, devoting himself to the sick crew, was unanimously elected a "member by invitation," and invited to take a seat on the platform. It was announced that Dr. Rose had left the city.

Dr. Francis G. Smith, of Philadelphia, chairman of the committee on publication, made his report showing the expense of publishing the annual volume.

Dr. Caspar Wistar, of Philadelphia, presented his annual report of receipts and expenditures, showing a balance on hand of $806. Accompanying the Treasurer's report was a resolution providing that the back volumes on hand, when over two years old, shall be sold at two dollars a volume, and that volumes V., VII., VIII., and IX., of which there are a surplus, be sold at $5 a set to any member.

The special committee on medical education, of which Dr. G. W. Norris, of Philadelphia, is chairman, were called upon to report. There was no response, and, on motion, the subject was referred to the committee on nominations.

Dr. A. B. Palmer, chairman of the committee on medical literature, asked leave to defer his report until Wednesday, at 10 o'clock, which was granted.

A report was made by the committee on nominations which was accepted, and the association then elected the following

OFFICERS:

President, Harvey Lindsley, of Washington city Vice Presidents, Drs. W. L. Sutton, of Kentucky; Thos. O. Edwards, of Iowa; Josiah Crosby, of New Hampshire; and W. C. Warden, of South Carolina. Secretary, Dr. A. J. Semmes, of Washington city. Treasurer, Caspar Wistar, of Philadelphia.

On motion, Drs. Flint, of New York, Gross of Pennsylvania, and Gibbs, of South Carolina, were appointed a committee to conduct the President elect to the chair.

Dr. Lindsley, having been introduced to the association by the retiring President, Dr. Eve, made a few pertinent remarks, acknowledging the honor as the highest he had ever been called upon to receive, and the highest that any medical man in America can receive. Unaccustomed to preside over so large a body, and having had but little practice in presiding over smaller assemblages, he must throw himself upon the forbearance of the association, and look to the members for support in the discharge of his official duties.

On motion, the thanks of the association were voted to the retiring officers for the able and impartial manner in which they have discharged the duties of their respective offices.

On motion, the ex-Presidents of the association present were invited to take seats on the platform.

The committee on medical topography and epidemics was called by States. A paper from the member from Maine stated that he will report next year. There was no response from New Hampshire, Vermont, Rhode Island, Connecticut or Massachusetts. Dr. Smith, of New Jersey, read an able report on New Jersey, and the association then adjourned until this morning at 9 o'clock.

*Second Day.*—The association was called to order by the President, Dr. Harvey Lindsley, and the Secretary read the minutes of the first day's proceedings, which were adopted.

On motion of Dr. Watson, of New York, Dr. Delafield, of New York, one of the first officers of the association, was invited to take a seat on the platform.

On motion of Dr. Atkinson, of Virginia, an amendment to the constitution was received, providing that no person shall be recognized as a member or admitted as a delegate at meetings of the association who has been expelled from any State or local medical association until relieved by action of such State or local association.

Dr. Atkinson supported the adoption of this amendment in an eloquent speech, contending that the admission of any gentleman who had been rebuked by any State or local association, and is under its ban, is a rebuke to that association. He urged the acceptance of the amendment, and trusted that until the constitution be so amended it shall be the rule of action.

Dr. Bond, of Maryland, asked to have the qualifications requisite for a seat read. He desired information as to the ethical qualifications for membership.

Dr. Watson, of New York, stated that, as by the constitution it was necessary to have amendments lie over one year, this was not a question for present debate.

The President decided that debate was not in order, and the amendment was accordingly laid on the table for consideration at the next annual meeting.

Dr. Boyle, of the committee of arrangements, proposed the names of Drs. Huff and Knight, who were elected "members by invitation."

Dr. A. B. Palmer, of Michigan, chairman of the committee on medical literature, made an able and interesting report.

REPORT ON MEDICAL LITERATURE.

After noticing in detail the periodical literature of the country, the spirit manifested in the editorial department of our medical journals is characterized as being (with a few exceptions) liberal, honorable, courteous and just; and the feelings of fraternity are generally cordial and warm. Differences of opinion must be expected occasionally to exist, and different interests will sometimes come in collision; and while this is the case, the imperfections of our common nature will be likely to produce some unpleasant results. But the bond of union produced by an interest in a common cause, and that cause so noble as the advancement of a great and benevolent profession, should certainly, as it usually does, smooth down asperities, and preserve that courtesy and kindness which ever should exist between gentlemen and brethren. From the contentions existing between the different portions of our common country, and which have so deeply affected the political, the religious and the literary periodicals, the medical journals, with scarcely an exception, have kept aloof; and it is devoutly to be hoped that the influence of this portion of the press, combined with the harmonizing power of this association, may ever be exerted for the promotion of union both of hearts and States. [Applause.]

The American medical literature of the past year was then reviewed, and said to have been of a creditable character, although it could not be denied that the fruits of the profession are more practical than scientific. The new American pharmaceutical association was noticed and complimented. The works auxiliary to medical science, issued by the federal government, were alluded to, and the example of the army and navy surgeons in taking the meteorological and other observations, commended to the brethren in civil life. The theses on the Parish will case were noticed as exhibiting the pre-eminence of American over British physicians. [Applause.] Prof. Agassiz and the support of his labors by the American public, came in for a share of praise, and several improvements in medical instruments were mentioned.

In closing his report, Dr. Palmer presented the following resume of the leading positions taken by the committee: The periodical literature of the United States is regarded as possessing great abundance, variety, richness and general excellence, and though still possessing defects, is constantly improving. Many of the contributions are of great weight and value, indicating an enterprising and industrious profession. Serious defects are regarded as existing in the review department, arising mainly from the fact that the income of the journals will not justify pecuniary disbursements for literary labor, and editors necessarily engaged in other pursuits cannot command the time, if all possessed the ability, to do the work thoroughly and well. [Applause.]

A few well supported journals, in place of the many but illy sustained, might tend to correct this evil; but the multiplicity of local journals is considered as peculiarly beneficial, by collecting from a greater variety of sources a larger number of facts, and developing the powers of a larger number of writers. The interests of this part of our literature demand a prompt and liberal pecuniary support.

The number of original American medical works is increasing, and their character is improving, and in some respects, particularly in practical utility, they will not suffer in comparison with those of Europe; yet serious imperfections exist, and great improvements are demanded. Great and permanent improvements in medical, as in general literature, must be gradual, depending more upon the advancement of education, of taste and intelligence, than upon any specific measures which may be adopted. Still, various particular measures, such as the permanent writing of medical theses during professional pupilage, and keeping systematic records of cases when in practice, would do very much in hastening an improvement. But for the greater perfection of our literature, we must wait the further development of our country, and for those changes of time and circumstances which shall produce a larger number of devoted savans and scholars, placing them in situations where a variety of absorbing pursuits shall not prevent the concentration of great talents upon a comparatively limited range of subjects.

On the subject of reprint of foreign works, it is held that, while the free circulation of the best class of these works among us increases the knowledge and improves the tastes of the masses of the profession, it does not interfere with the production of the higher order of original works; and that the moral obligation of our government to join with Great Britain in the enactment of an international copy-right law, is by no means established.

In conclusion, the committee would say that if, as sentinels placed upon the walls of our medical Zion, they are asked in relation to its literature, "What of the night?" the response must be, "The morning cometh!" The darkness which has hung over that literature is breaking away. There is at least dawning in the East, and though the chariot of a day may roll on but slowly, the full effulgence will come at last. [Continued applause.]

On motion, the report was accepted, and ordered to be published.

On motion, Dr. Bozman, of Alabama, was elected a "member by invitation."

REPORT ON MEDICAL EDUCATION.

Dr. James R. Wood, chairman of a special committee on medical education, made a lengthy report, discussing: 1st, primary medical schools; 2d, the number of professorships in medical colleges; 3d, the length and number of terms during the year; 4th, the requisite qualifications for graduation; 5th, such other subjects of a general character as to give uniformity to our medical system. Having reviewed these propositions at length, the committee have arrived at the following conclusions:

*First.*—Primary medical schools should be encouraged; but, as office instruction will continue to be sought by students, practitioners should either give them necessary advantages of demonstrations, illustrations, and recitations, or if not prepared to do so, they should refer them to such primary schools, or medical men, as will give them proper instruction.

*Second.*—The number of professorships should not be less than seven, viz: a Professor of Anatomy and Microscopy, Physiology and Pathology, Chemistry, Surgery, Practical Medicine, Obstetrics, and Materia Medica.

*Third.*—There should be but one term annually, which should commence on the 1st of October, and close with the March following, thus lengthening the term to six months. The commencement of the term, in October, should be uniform in all the colleges throughout the country. During the session there should never be more than four lectures given daily.

*Fourth.*—The qualifications for graduation, in addition to those now required by the schools, should be a liberal primary education, and attendance upon a course of clinical instruction in a regularly organized hospital.

In order to give our medical colleges an opportunity to consider the recommendations here advanced, and that this body may have the advantages of their wisdom and their mature views, before any definite action is taken upon them, your committee submit to the association the following resolutions:

*Resolved*, That the several medical colleges of the United States be requested to send delegates to a convention to be held at on the day of for the purpose of devising a uniform system of medical education.

*Resolved*, That the report of the special committee on medical education be referred to such convention for its consideration.

*Resolved*, That said convention of delegates from the several colleges of the United States be requested to submit to the meeting of this association in May, 1859, the result of their deliberations.

On motion, the report was accepted and referred to the committee on publication, the accompanying resolution being laid on the table.

The committee on nominations reported Louisville, Ky., as the place of meeting in 1859, and nominated Dr. S. M. Bemis, of that place, as second Secretary. They also nominated the following standing committees:

*Committee on Publication.*—Dr. F. Gurney Smith, Pa., chairman; Drs. Caspar Wistar, Pa., A. J. Semmes, D. C., S. M. Bemis, Ky., S. L. Hollingsworth, Pa., S. Lewis, Pa., H. F. Askew, Del.

*Committee on Medical Literature.*—Dr. J. Watson, N. Y., chairman; Drs. L. A. Smith, N. J., C. G. Comegys, Ohio, R. W. Gibbs, S. C., W. M. McPheeters, Mo.

*Committee on Prize Essays.*—Dr. J. B. Flint, Ky., chairman; Drs. M. Goldsmith, N. J., H. Miller. Ky., Calvin West, Indiana.

*Committee on Medical Education.*—Dr. G. W. Norris, Pa., chairman; Drs. A. H. Luce, Ill., E. Henderson, S. C., G. R. Grant, Tenn., T. S. Powell, Ga.

*Committee of Arrangements.*—R. J. Breckinridge, Ky., chairman; Drs. G. W. Ronald, B. M. Wible, D. W. Goodall, D. D. Thompson, N. B. Marshall, G. W. Burglass, R. C. Hewett, and A. B. Cook, all of Kentucky.

The report was accepted, the nominations were confirmed, and the committee received permission to sit again.

On motion of Dr. Hamilton, of Buffalo, the resolutions attached to the report of the committee on medical education were taken from the table.

Dr. Watson moved the appointment of a committee to consider the resolutions and report to-morrow morning.

Dr. Bond thought that the subject had already been sufficiently discussed. It had been brought up year after year, occupying much of the time of the association, and he trusted that it would receive immediate consideration.

Dr. Davis, of Illinois, wished to have the subject made a special order for some time prior to the adjournment of the convention.

Dr. Rogers, of New York, wished to have the report printed, that all might have an opportunity of examining it and the propositions which it embodies.

Dr. Wood defined his report as a conservative report, just alike to the profession and to the laymen. He did not believe that any good could arise from a further discussion of the subject. None has arisen in years past—none could arise now. It was a bill of conciliation and adjustment. Laymen of the profession merited censure for sending men to college not qualified for the profession, and colleges merited censure for sending men out not qualified to practice the healing art. He approved of the motion of Dr. Watson, that the report be submitted to a committee of delegates from colleges.

A debate on a call for a previous question on Dr. Watson's resolution then ensued, in which several gentlemen joined, each one appparently having a different idea of "parliamentary law," and neither of them displaying a very correct knowledge of the subject. It was remarked by an old member of the association, that "parliamentary discussion must be a *local epidemic.*"

The report was finally referred to a select committee, to be composed of one member from each delegation representing a medical college or school.

On motion, thanks were voted to the late Secretary, Dr. Foster, and his successor, Dr. Bemis, took his seat.

Dr. Hamm, of Pennsylvania, moved a suspension of the rules for the purpose of reconsidering the resolution of Dr. Condie, accepting the apology tendered by Dr. Bryan. The vote upon suspending the rules stood—ayes 111, nays 82. The President ruled that a two-thirds vote was necessary, and decided the question as lost.

An appeal was taken from the decision of the chair, and the decision was not sustained. A vote was then taken, and the resolution accepting the apology of Dr. Bryan was reconsidered by a vote of—yeas 142, nays 70.

An attempt was then made to connect the resolution with that accepting the apology of Dr. Reese, but it was decided that it would first, be necessary to dispose of the resolution reconsidered, and it was laid on the table.

A member from New Jersey hoped that the McClintock case would be brought fairly and squarely before the association, and that gentleman would be made to "face the music." It was useless to cloak it, or to attempt to dodge the responsibility.

Dr. Beck, of Indiana, moved an indefinite postponement of the whole subject.

Other gentlemen rose to speak, but the President decided that a motion to postpone was not debatable.

Dr. Jewell rose to a point of order, and protested against being "gagged." [The President here reversed his decision.] Dr. Jewell said that the action of the day previous was regretted, and that gentlemen had acted hastily. Many, who voted at first sight to accept the apologies, now regretted having done so.

Dr. Hamm, of Philadelphia, explained the action of the Philadelphia County Medical Society, and began to read a remonstrance from it, which he desired to incorporate into his speech.

Dr. Biddle objected to the reading of this remonstrance, as a violation of plighted faith.

It was here moved and decided that the association go into "committee on the whole," and Dr. Edwards, of Ohio, was called to the chair.

A member hoped that there would be no rules of order except what the chair would prescribe.

The Chair: "I will prescribe enough." [Laughter.]

Another member enquired if it would be proper to discuss the remonstrance?

The Chair: "A gentleman who has the floor can discuss anything on the face of the earth." [Laughter and applause.]

The remonstrance was then read. It was a long document, giving a detailed account of the recommendation by Dr. Reese of Dr. McClintock for a position in Blockley hospital, after the last named gentleman had been guilty of selling quack nostrums, and had thus committed an offence against the ethics of the profession.

Dr. Humphries, of Indiana, moved that each member of the committee of the whole be restricted to five minutes, allowing Dr. Reese whatever time he wished to defend himself in.

Dr. Phelps showed that a ten minutes rule was now in force.

Dr. Cox moved, as an amendment, to make the time fifteen minutes, which amendment was lost, and the original motion of Dr. Humphries was then carried.

Dr. Reese then ascended the platform, and made a statement of his position from the commencement of the controversy. He considered his apology of the day previous a satisfactory one, but was willing to make it more so if it was objected to. He had not brought the subject before the association, but had been given to understand that if he made the apology which he had made the remonstrance would not be offered. During his remarks there was a demand for the reading of the apology; which was read, as follows:

*To the Officers and Members of the American Medical Association.*—The undersigned, one of the vice-presidents of the American medical association, having, during the interval since our last annual meeting, certified to the professional fitness for the charge of the Blockley Hospital at Philadelphia, of an individual who had been expelled from this body for a violation of our code of ethics, after consultation with the officers, and yielding to the advice of other personal friends, desires to say to the association now assembled:

1st. That, in giving said certificate, he was prompted solely by motives of sympathy and humanity to a fallen brother, who had been a personal friend prior to his offence; and that he did not realize, acting under the impulse of the moment, that his individual act could be construed by the profession as indicating hostility to his brethren.

2d. That while his own mind is clear that his certificate contained only the truth, and that, under his peculiar relations to the party concerned, he could not withhold his certificate of medical qualification, consistent with conscience and duty, yet he is ready to concede that he had no abstract right to relieve the party from the censure of the association until this body had restored him to his fellowship.

3d. That, so far from intending any disrespect to the association or to its acts of discipline, the undersigned had publicly sustained and defended both. He therefore disclaims the inference from his certificate that he intended to recommend to a high professional office a man whom the association had excluded, and thereby nullify the action of this body.

And, finally, with these statements and disclaimers, the undersigned, while retaining his own opinion of the rectitude of his motives, and of his duty, under the peculiar circumstances of the case, is nevertheless prepared to defer to the judgment of those whom he knows to be his friends, that he erred in doing what he had no right to do, in view of his official position in the association, and is hence called upon to offer this explanation and apology to his brethren.

Signed, DAVID M. REESE.

It was moved to refer the apology and remarks of Dr. Reese to a special committee of seven, to report to-morrow morning. Dr. Atlee, of Lancaster, and other gentlemen urged delay.

Dr. Payne of Virginia, asked permission to relate an anecdote. He was reminded of two old Quakers, one of whom kept a store, while the other practiced law. Both were members of a temperance society, and it was thought that the lawyer did not always keep his pledge. One wet, cold day, a negro went to the Quaker's store, and the good man gave him a drink of brandy. This was brought to the notice of the temperance society, and it was decided that the defendant should be severely reprimanded. The lawyer was selected to carry out this sentence, and, taking the store-keeper into the woods, he thus addressed him: "Jeemes, thee should be more circumspect!" [Continued laughter.]

Dr. Condie, of Philadelphia, wished to say that he had offered the resolution in good faith, but he denied that he had made propositions to the gentleman from New York, or that the Philadelphia committee had.

Dr. Bowling, of Tennessee, said that there was no question of veracity. Gentlemen on either side were correct. He had heard of misunderstanding and of probable difficulty, and had earnestly endeavored to arrange it. He had told Dr. Reese that if he made an apology, the remonstrance would not be presented, because he had understood gentlemen from Pennsylvania to say so. But he was now aware that these gentlemen did not in any way pledge to the Philadelphia county medical society.

Dr. Condie hoped that a committee would be appointed to give the subject a careful consideration.

Dr. Cox, of Maryland, after complimenting Dr. Reese as an able practitioner and an experienced editor, whose labors have been of great value to the profession and to the country, said that he did not consider the statement full and satisfactory. The offence was not an unpardonable one, but the violation of that code of ethics which is the life of the profession, should be properly atoned for. [Applause.] The apology was good enough, but it carried as its sting the mental reservation which Dr. R. persists in. Nay, in his journal, issued simultaneously with this meeting, and circulated here, he says: "Having done right in certifying to the labors of our quondam friend McClintock, we resented the unmerited censures of our Philadelphia brethren." This completely stultified the effect of the apology.

Dr. La Roche, of Philadelphia, explained his action and that of the Philadelphia county society in the matter.

Dr. Payne of Vermont, and Drs. Cox and Bond made some rather sharp remarks. Dr. Davis, of Massachusetts, thought that Dr. Reese had but to admit that he had done wrong, and ask pardon, without any mental reservation.

Dr. Reese said that he had intended to make a satisfactory apology. Such was his earnest wish and desire, and he wished to frankly state that he had no mental reservation, neither did he attempt to conceal anything. He made the statement which had been read without reservation and without evasion. [Applause.]

Dr. Condie expressed his entire satisfaction, as did numerous other gentlemen, several crossing to where Dr. Reese was sitting and shaking hands with him.

The committee of the whole then rose, and the chairman reported to the president that the committee had heard and discussed the apology of Dr. Reese, and that they considered that it was "ample, full, complete and satisfactory."

On motion, the report of the committee was received and adopted.

The case of Dr. Bryan then came up, when it was suggested that his apology should be in writing, he expressing a willingness to make one as ample as was that of Dr. Reese.

Dr. Reese then drafted an apology, but several gentlemen insisted that he should insert the word "regret." Dr. Reese declined, stating that no gentlemen would apologize for what he did not regret, and that he would never be dictated to by any gentleman, even if the prison door stood open on his right hand, and the stake was at his left hand.

Dr. Wood (who was greeted with loud applause) stated that he had been with the side which offered the apology, but he did not consider the apology complete without the insertion of the word "regret."

Drs. Bonner, Clark of New Jersey, Hard of Illinois, Parker of New York, and other gentlemen participated in an exciting debate on the necessity of having the word "regret" inserted.

Dr. Reese added the following sentence, "and regrets that he has incurred the displeasure of his brethren." This was not favorably received.

Dr. Boyle, chairman of the committee of arrangements, here announced that arrangements had been made by which the delegates who had purchased tickets on their way to the convention over the following roads, could return free by exhibiting their cards of membership: Pennsylvania, Wilmington and Manchester, Illinois Central, Northeastern South Carolina, and Richmond and Petersburg.

The apology of Dr. Reese was again taken up, and discussed with spirit, although there was no manifestation of bad feeling on either side. At length he presented the following:

"The undersigned regrets that he certified to the professional qualifications for Blockley hospital, Philadelphia, of an expelled member of this body, and hereby offers this apology for his departure from the ethical code."

This was received with loud applause, and, on motion of Dr. White, accepted as an ample and satisfactory apology.

Dr. Bryan then submited a similar apology, which was also accepted, and then the committee adjourned until to-morrow morning, 9 o'clock, evidently well pleased that this question was finally disposed of.

---

*Washington, May* 6, 1858.—The president, Dr. Lindsley, called the association to order at half-past 9 o'clock. The secretary not having his minutes in readiness, the reading of the minutes of the day previous was dispensed with.

Dr. Grant, of New York, asked leave to present a complaint against the New York medical college; but upon information by Dr. Edwards that a committee on ethics would be recommended by the nominating committee, he withdrew his request.

The minutes were then read. Several proposals to amend them were made, and either ruled out of order or withdrawn.

The appointment during the last year of Dr. Geo. Haywood, of Boston, as a delegate to represent the American medical association in kindred societies in Europe, was announced by Dr. Eve.

MEDICAL EDUCATION.

Dr. Hamilton, from the committee of delegates from the medical schools and colleges, to whom was referred the report of the special committee on medical colleges, reported the following preamble and resolution:

Fully appreciating the value and importance of the resolution under which they were appointed, but a majority of the gentlemen constituting this committee not being authorized by the medical faculties of the several colleges with which we are connected to act as their representatives in this matter, and therefore regarding it quite impossible to secure a convention of delegates in the interim of the meetings of the association: therefore—

*Resolved*, That we recommend to all the medical colleges entitled to a representation in this body, that they appoint delegates, especially instructed to represent them in a meeting to be held at Louisville, on Monday, the day immediately preceding the convention of the American medical association for the year 1859, at 10 o'clock, at such place as the committee of arrangements shall designate.

The report was accepted, and the preamble and resolutions were passed; after which several gentlemen claimed the floor, but the president decided that reports of special committees were in order, the reports of committees on medical topography and epidemics having previously been referred to the committee on publicaton without reading.

Dr. Foster Jenkins, of New York, read a report on the spontaneous umbilical hemorrhage of the newly born, which was read and referred to the committee on publication.

MARRIAGES OF CONSANGUINITY.

Dr. S. M. Bemis, of Kentucky, read an able and learned report on the "influence of marriages of consanguinity upon offspring," from which we extract the following valuable statistical information:

Your reporter has made great effort to ascertain the proximate per centage of the deaf and dumb and blind in our asylums who are the descendants of blood intermarriages. This effort has not been successful, from the difficulty principals of such institutions find in gaining the requisite facts. Parents are often sensitive on this score, and it is a delicate matter for principals to attempt investigations which the friends of the beneficiaries suppose to be unauthorized by the regulations of their various institutions.

I feel, however, that my researches give me authority to say, that over ten per cent. of the deaf and dumb, and over five per cent. of the blind, and near fifteen per cent. of the idiotic in our State institutions for subjects of those defects, are the offspring of kindred parents.

Aside from the facts which I have gained by corresponding with gentlemen who have given close attention to these points, a curious but perfectly legitimate process of computation confirms me in the opinion that those estimates are very nearly correct. The classes C, D, E, F, G give 787 marriages of cousins, 246 of which have given issue to deaf and dumb, blind, idiotic or insane children. Admitting the same ratio to prevail, the Ohio report, which contains 157 marriages of cousins, followed by deaf and dumb, blind, idiotic or insane offspring, would indicate the existence of 332 other marriages of cousins in the same population not followed by such defects. The counties which furnish this 151 marriages as above, and are supposed to comprise in their limits 332 unreported marriages, making a total of 483, contained in 1850 a population of 1,528,238. If the same ratio be supposed to exist throughout the Union, there would be found to the twenty millions of white inhabitants six thousand three hundred and twenty-three marriages of cousins, giving birth to 3,909 deaf and dumb, blind, idiotic and insane children, distributed as follows:

| | |
|---|---|
| Deaf and dumb, - - - - - - | 1,116 |
| Blind, - - - - - - - | 648 |
| Idiotic, - - - - - - - | 1,854 |
| Insane, - - - - - - - | 299 |

Then, if the figures of our last United States census still applied to our population, there would now be found in the Union:

9,136 deaf and dumb, of whom 1,116, or 12.8 per cent. are children of cousins.

7,978 blind, of whom 648, or 08.1 per cent. are children of cousins.

14,257 idiotic, of whom 1844, or 12.93 per cent. are children of cousins.

14,972 insane, of whom 299, or 01.9 per cent. are children of cousins.

I invite the attention of gentlemen of this association to this calculation of probabilities, either to confute or confirm it by any facts in their possession.

A very cursory examination of the tables of my report will suffice to show that pari passu with the increment of the same blood the sum of defects of offspring is likewise increased. Classes D and G present exceptions to this rule. As it regards

D, the supposed reason for this deviation has been already stated, namely, that its mortality list is so large; while class G presents so few observations as to satisfy us that they attracted notice solely because of their unfavorable results.

STONE CONTRIBUTED TO THE WASHINGTON MONUMENT.

Dr. John L. Atlee, from the committee appointed at the annual meeting at Richmond in May, 1852, to procure a stone with a suitable inscription to be inserted in the Washington national monument, made a final report. It stated that Mr. Haldy, a marble mason of the city of Lancaster, Pennsylvania, had in his employment a young man, Mr. J. Augustus Beck, a native of Leitz, Pennsylvania, who had given unmistakable evidence of his genius as a sculptor. At the suggestion of the late lamented Dr. A. L. Pierson of Salem, Massachusetts, (made at the meeting in New York just ten days before his death), the design of the celebrated painting of Girodet-Tricoson, representing Hippocrates refusing the presents of the Persian King, Artaxerxes, and his invitation to leave Greece, and reside and practice among her enemies, was selected. This was sculptured upon a block of Vermont marble, with the motto, "Vincet Amor Patriæ," and the stone is now at the monument grounds. The entire expense was $1,000, of which one-half was paid to the young artist. The amount contributed by members individually was $501 30; the balance was voted from the treasury of the society. Accompanying the report was a letter from the secretary of the Washington national monument association, and a resolution of thanks to the rail road companies by whose liberality the stone was brought from Lancaster to Washington free of charge. The report was accepted, and the resolution was passed.

REPORT ON THE FUNCTIONS OF THE CEREBELLUM.

Dr. Palmer of Buffalo read a report, made by Dr. E. Andrews of Chicago, Illinois, on the "functions of different portions of the cerebellum."

Dr. Campbell of Georgia read a report on the "nervous concomitants of febrile diseases;" which was accepted and referred to the committee on publication.

Dr. J. Marion Simms of New York city read an abstract of his report on the treatment and the results of obstructed labor, illustrated with a series of magnified illustrations. The doctor seemed perfectly familiar with his subject, and was frequently applauded.

Dr. Stephenson of New York read an interesting abstract of his report on "the treatment best adapted to each variety of cataract, with the method of operation, place of selection, time, age, &c." Dr. Stephenson is the surgical director of the New York opthalmic hospital, an institution which relieves hundreds every year from sufferings with diseases of the eye, besides enabling pupils to receive invaluable instructions.

On motion, other reports were called for, read by their titles, and referred to the committee on publication.

COMMITTEES FOR THE ENSUING YEAR.

Dr. Edwards, from the committee of nomination, offered the following list of committees for the ensuing year, which was accepted, and the committees were chosen:

*Special Committee on the Microscope.*—Drs. Holsten of Ohio, Dalton of New York, Hutchinson of Indiana, Stout of California, and Ellis of Massachusetts.

*Special Committee on Medical Jurisprudence.*—Drs. Smith of New York, Hamilton of Buffalo, Crosby of New Hampshire, Purple of New York, and Mulford of New Jersey.

*Committee on Quarantine.*—Drs. Harris of New York, Moriarty of Massachusetts, La Roche of Pennsylvania, Wragg of South Carolina, and Fenner of St. Louis.

*Committee on Surgical Pathology.*—Dr. James R. Wood of New York, chairman.

*Committee on Diseases and Mortality of Boarding Schools.* Dr. C. P. Mallengly of Kentucky, chairman.

*Committee on the various Surgical Operation for the Relief of Defective Vision.*—Dr. Montrose A. Pallen of St. Louis, chairman.

*Committee on Milk Sickness.*—Dr. Edward A. Murphy of Indiana, chairman.

*Committee on Medical Ethics.*—Drs. John Watson of New York, Dalton of Massachusetts, Emerson of Pennsylvania, Hamilton of New York, and Gaillard of S. Carolina.

RESTORATION OF DR. BAILEY.

Dr. Edwards also reported from the committee of nomination the following resolution:

*Resolved*, That a committee of nine be appointed by the chair to wait on the Hon. Howell Cobb, secretary of the treasury, and respectfully request the restoration of Dr. M. J. Bailey as inspector of drugs and medicines for the port of New York.

Dr. Edwards, in an elaborate and eloquent argument, urged the adoption of this resolution.

Dr. Tyler of Georgetown rose to reply, but gave way to permit Dr. Condie to have some special committees on medical subjects appointed.

Dr. Palmer moved to adopt the appointment of these committees as recommended, which was carried.

Dr. Tyler said that there was no member of the medical profession in the country who felt more indebted than himself to

Dr. Edwards for his agency in procuring the passage of a law for the inspection of drugs and medicines. [Applause.] It was unnecessary to say a word in regard to the benefit which has resulted from the passage of that law, nor did he wish to be misunderstood in his opposition to the resolution. But when it was proposed to appoint a committee to wait upon an executive officer of government, and dictate to him, he felt that it would be turning aside from the purposes for which this association was organized.

The gentleman who introduced the resolution had said that Dr. Bailey would not have turned from his laboratory to elect a president. He commended him for it. But he would not have this association leave its noble sphere of action to approve or denounce an appointment avowedly made upon political grounds. [Applause.] If the association leaves the field for which it was organized, and in which it has steadily labored for eleven years, he felt confident that it would result in no good—it might result in injury. He had not anticipated this action. He had given the subject no consideration ; but it struck him as directly in opposition to the prosperity and the popularity of the association, and he asked gentlemen to pause ere they voted for the resolution.

Dr. Bolton of Virginia urged the adoption of the resolution.

Dr. Cox of Maryland concurred with Dr. Tyler in acknowledging the value of the services of Dr. Edwards, and he also concurred with him in objecting to the political, personal and special character of the resolution which that gentleman had presented. The passage of such a resolution would open the door for unpleasant action hereafter. If a change was recommended at New York, one would be recommended at Boston, at Philadelphia, and at Charlestown. That it would be well to disconnect those offices from politics, he would admit ; and he would consequently offer the following resolution as a substitute for that of Dr. Edwards :

Resolved, that the appointment of inspectors of drugs and medicines in the various ports of the United States should, in the opinion of this association, have regard to the essential, moral and scientific qualifications of the candidates, and not to considerations of personal favoritism or political bias.

Dr. Edwards trusted that the substitute would not be received by the association. The present occupant of the post of New York was notoriously unqualified for it. He wondered that gentlemen should object to seeing him displaced to make room for the reinstation of a gentleman whose qualifications no one could question. Nor did he think that the secretary of the treasury would consider it an interference if the representatives of the medical profession of the whole country made a suggestion to him. Besides, Dr. Bailey is a democrat, therefore the removal is not a political one. He had reason to believe that upon a proper suggestion, Dr. Bailey would be restored. If he was not restored, the law would be repealed before the close of the present session.

Dr. Tyler supported the substitute offered by Dr. Cox, as a fair compromise. He believed that the appointment of a committee would transform the association into a political machine. He honored the gentleman from Ohio for the evidences of a warm heart which he had exhibited in endeavoring to procure the restoration of a friend to office, but with him a great principle was at stake. Gentlemen who do not reside in this district may not understand how the heads of departments are importuned about the offices, and how jealously the motives of all who take part in the appointment or the removal of office holders are scanned. He should prefer to see the resolution of Dr. Cox passed.

Dr. Dunbar enquired if it was the duty of a committee of nomination to nominate a candidate for inspector general of drugs at New York. [Laughter.]

Dr. Batchelder of New York called for the reading of the original resolution presented by Dr. Edwards.

Dr. Parker of Virginia offered other resolutions as a substitute for those offered, which were lost.

The question was then taken on accepting the substitute offered by Dr. Cox, which was lost.

Dr. Wilcox of Connecticut offered an amendment to the resolution of Dr. Edwards, "disclaiming all political considerations." The amendment was accepted by Dr. Edwards.

Dr. Batchelder testified to the qualifications of Dr. Bailey, whom he had known from his pupilage.

Dr. Jewell of Philadelphia hoped that the resolution would not pass. If it did, he would ask to have a gentleman at Philadelphia removed. Boston members will do the same, and this association will be wholly occupied with offices.

Dr. Wood of New York said that he rose from a sense of duty, and frankly confessed that he should vote contrary to his personal predilections, which were in favor of Dr. Bailey. [Applause.] But an endorsement of him would be a bad precedent. If we are to make ourselves judges of any individual, we must make ourselves judges of all individuals. If we sit in judgment on the gentleman now holding this office, we shall gradually sink into political partizanship, and lose our present high position. He could not consent to sanction the public action of the association, although if a petition was drawn up for the reinstation of

Dr. Bailey he would be pleased to see it signed by every member present in his individual capacity.

Dr. Rodgers of New York said that the subject had not been brought before the association at the recommendation of the delegation from New York, and he moved that it be laid on the table.

The previous question was called and sustained. The motion to lay on the table was defeated—ayes 49, nays 64.

Dr. Sayer of New York eulogized Dr. Bailey as eminently fitted for the place, and condemned the present incumbent.

The resolution as amended was then carried by a vote of 79 ayes to 52 nays.

Resolved, that a committee of nine be appointed by the chair to wait on the Hon. Howell Cobb, secretary of the treasury, and respectfully to request the restoration of Dr. M. J. Bailey as inspector of drugs and medicines for the port of New York—at the same time disclaiming all political considerations.

Dr. Bohrer of Georgetown, chairman of the committee on special medical essays, stated that they had not had time to read, much less consider, the papers placed in their hands.

On motion, the committee on special medical essays was instructed to hand such papers as they deemed worthy, to the committee on publication.

The president announced, as a special committee to wait on the secretary of the treasury, Drs. Arnold of Georgia, Atkins of Virginia, Buckley of New York, Hayes of Pennsylvania, Smith of New Jersey, McPheeters of Missouri, Hargraves of Alabama, Richter of Michigan, and Hooker of New York.

On motion, Dr. Edwards was added to the committee as chairman. He declined, giving personal reasons as an excuse; but the committee refused to accept it, and he was accordingly chosen.

A gentleman stated that he, with several friends, had voted for the resolution with the sole intention of moving its reconsideration.

Dr. Grant of New Jersey presented a complaint made by the Newark medical society against the Medical institute of N. York, for a violation of the ethics of the profession. Dr. Edwards presented a similar complaint, and Dr. Oakley, a complaint from the Union and Essex county medical societies. They were received and referred.

Dr. Sutton of Kentucky moved that Dr. Jarvis of Massachusetts have further time to report on a uniform system of registration of births, marriages and deaths, and that a committee be appointed to urge upon the census bureau of 1860 the importance of having a physician attached to it to collect vital statistics.

Dr. Kyle of Ohio proposed an amendment to the constitution, by which no person can sit as a member or a delegate at meetings of this association, who is not a graduate of a recognized medical college. Laid over for one year, under the rules.

Dr. L. A. Smith presented resolutions of the New Jersey medical society, praying for such changes of the constitution as would establish a board of census in every judicial circuit of the supreme court, who should examine and grant diplomas to all proper members of this association. Laid over for one year, under the rules.

Dr. Humphries of Indiana presented a resolution praying for an interchange of transactions of state and county societies, which was adopted.

Dr. Boyle, chairman of the committee of arrangements, presented the names of Prof. Swallow of Missouri, and Prof. Mittag, as "members by invitation," and they were elected.

An invitation from Prof. Bache to visit the coast survey bureaux, on Capitol hill, was read, accepted, and a vote of thanks for the courtesy was passed.

Dr. Gibbs of South Carolina moved that Professor Henry be requested to favor the association with his views on meteorology, at such time during the session as he may select.

Carried unanimously.

Dr. Campbell of Georgia moved that the secretary place on record an expression of regret with which the society has learned the death of Drs. C. R. Walton, S. W. Granton, Marshall Hall, T. Y. Simmons, Mitchell, and other members deceased since the last annual session. Carried.

VOTE OF THANKS.

On motion of Dr. Phelps, the following resolutions were passed unanimously, the members rising.

Resolved, that the thanks of this association are eminently due to the regents and Prof. Henry, of the Smithsonian institution, for the ample and convenient accommodation afforded for the transaction of business.

Resolved, that the committe of arrangements are entitled to our praise and highest appreciation of their exertions to promote the comfort of the members and the best interests of the association.

Resolved, that to the physicians of Washington and Georgetown and the faculty of Georgetown college we accord the homage of our sincerest thanks for their elegant hospitalities extended to the members from abroad, by which the pleasure of their sojourn here has been so greatly enhanced.

Resolved, that we feel assured that the impressions on the tablet of memory received here, in our national metropolis, in this the first year of the second decade of the association, will long remain an evidence of the urbane attentions received not only from the chief magistrate and other public functionaries of our glorious Union, but of private citizens and the community at large.

Resolved, that the manifestations of union of heart and purpose in the action of this session inaugurate a new era, and call for devout acknowledgment of Divine Providence, and presage, as we trust, not only a bright future for the association, but also as contributing to the perpetuity and prosperity of our great national confederation.

On motion of Dr. Anderson of New Jersey, it was unanimously resolved that the thanks of the medical association be presented to Rev. Dr. McGuire and his faculty of the College of Georgetown, for their very cordial reception and entertainment of the association at the college yesterday.

Dr. Arnold of Georgia then exhibited specimens of a new method of medical preparation of some membrane incomprehensible to the reporter, but which was evidently very interesting to the association.

On motion of Dr. Foster of Tennessee, it was resolved that after 1860 Dr. Hamilton have the privilege of using his report on "deformities after fracture," published in the Transactions, for a work which he proposes to publish.

Dr. Duhamel of Washington moved that a committee be appointed to investigate and report upon the "National Hotel disease."

Dr. Foster of Tennessee opposed the appointment of such a committee, as did Dr. Boyle of Washington. Dr. Duhamel withdrew his motion.

Dr. Gaston of South Carolina exhibited a new instrument of a novel construction.

Dr. Campbell of Georgia was not aware, until he had just heard permission granted to Dr. Hamilton, that he had transgressed in republishing in a work a report which he had contributed to the Transactions of the association. [Cries of "Regret it," "regret it."] He did regret it, and asked the sanction of the society, which was granted.

The president appointed Drs. Miller, Antisel, and Garnet a committee to wait on the census bureau, as provided by the resolution of Dr. Sutton.

Dr. Dunbar moved a reconsideration of the vote appointing a committee to request the reinstation of Dr. Baily, and Dr. Morgan seconded it, but, as Dr. Parker had been invited upon the platform, the motion was ruled out of order.

DR. PARKER'S CHINESE HOSPITAL.

Dr. Peter Parker, ex-commissioner to China, was then introduced, and was received with applause. He exhibited some curious specimens of calculi, as the results of thirty-eight operations upon the Chinese. They were of various shapes and composition, and weighed from a few drachms up to three, seven and eight ounces. His description of the operation by which these calculi were removed was deeply interesting, and it was gratifying to learn that out of the thirty-eight patients, all but five or six recovered perfect health.

Dr. Parker proceeded to state that he had treated in China, at the hospital under his charge, fifty-three thousand cases. Pictures of the most curious cases he had brought to this country, and they were on exhibition in the room below. At no very distant period he hopes to place in a permanent form the result of his labors, with illustrations. [Applause.] Among other cases, he had probably performed upwards of a thousand operations for cataract. One day he operated in sixteen cases, the youngest being a mere child, and the oldest, an old lady seventy-nine years of age. She came, led by a servant, submitted heroically to operations on both eyes the same day, and in a fortnight had her sight perfectly restored. [Applause.] In acknowledging a vote of thanks, Dr. Parker said he had among his patients all classes, from members of the imperial family down to beggars. His greatest difficulty had been to persuade his patients that he could not cure all diseases.

RECONSIDERATION OF THE DR. BAILEY RESOLUTION.

Dr. Dunbar claimed the floor, and urged the reconsideration of the vote appointing a committee to wait on the secretary of the treasury, and solicit the reinstation of Dr. Bailey.

Dr. Payne of Virginia opposed the reconsideration.

Dr. Tyler advocated it, and asked if this association was formed to wait on executive officers, and to dictate to them who they shall remove and who they shall appoint. Many gentlemen, around him, he was assured had voted for the resolution without due reflection, and he trusted with confidence in their sober second thought. [Applause.] The press and the profession, he felt confident, would denounce this association if it entered into the wide field of politics. It was instituted to promote the great cause of science, not to join issue with government. [Applause.]

Dr. Morgan also advocated a reconsideration. He was not a partizan. Although he resides in Washington, he has no personal acquaintance with the president or the secretary of the treasury, but he was confident that they would not have made the change without good reason, and it was not the mission of this association to criticise or to attempt to change their views.

Dr. Palmer, after stating how little regard he had for the opinions of the press, enquired as to the present incumbent of the office. Is he capable?

Dr. Watson of New York said that Dr. Bailey had had his circulars out since his "rotation," and the subject had been twice before the academy of medicine, who have ignored it.

Dr. Burns of Brooklyn said that he was not a politician, and that he was a personal friend of Dr. Bailey, but he hoped that the vote would be reconsidered.

A member from California related his experience there on a question as to the superintendent of a lunatic asylum. In his opinion the less the association had to do with politics, or with expressions of opinion on political appointments, the better. [Applause.]

Dr. McNulty of New York said that the question had been twice before the New York academy of medicine, and twice been voted down. The present incumbent, whom it is sought to oust, is a German by birth and education. He can read the invoices in whatever European language they may be sent, and he makes his own analyses, which it is reported the ex-inspector did not do.

After some "parliamentary" skirmishing, it was decided to reconsider by a vote of 51 ayes to 32 nays. And, on motion, the subject was then indefinitely postponed.

The association then took a recess of two hours, for dinner.

EVENING AND CLOSING SESSION.

The association was called to order at 5 o'clock, P. M., by Dr. Sutton, one of the vice presidents, who took the chair.

The amendments to the constitution, proposed at the annual meeting at Nashville, had been made the "special order." They were—

1st. Amend the third article of the constitution, in relation to meetings, by inserting after the words "first Tuesday in May," the words "or the first Tuesday in June;" and also inserting after the words "shall be determined," the words "with the time of meeting."

2d. In article 2, omit the words "medical colleges," and also the words "the faculty of every regularly constituted medical college, or chartered school of medicine, shall have the privilege of sending two delegates."

Each amendment was separately discussed, and each was lost by a large vote. An amendment proposed at Philadelphia in 1856, providing for the establishment of a permanent secretaryship, was lost by a vote of 53 ayes to 84 nays.

On motion of Dr. Foster of Tennessee, the secretary was directed to collect all the by-laws, and have them printed in the next volume.

An attempt was made to introduce a motion endorsing the accoustics and ventilation of the new capitol extensions, but it was ridiculed by Dr. Sayer, and withdrawn.

Various additional votes of thanks were passed, and, at ten minutes of seven, the association adjourned *sine die.*

